# Benefits of Combined Therapies in Burn Lesions: Enzymatic Debridement and Other Modern Approaches—Our Clinical Experience

**DOI:** 10.3390/life15030352

**Published:** 2025-02-24

**Authors:** Angela Tecuceanu, Camelia Tamaş, Anca Sava, Ruxandra Vatavu, Andreea Mioara Avram, Iulia Olaru, Bogdan Mihnea Ciuntu, Irina Mihaela Abdulan, Roxana Ciuntu, Mihaela Corlade, Irina Mihaela Hreniuc Jemnoschi, Andreea Ludușanu, Irina Bușilă, Teodor Stamate, Cristinel Ionel Stan

**Affiliations:** 1 Department of Morphofunctional Sciences I, “Grigore T. Popa” University of Medicine and Pharmacy, 700115 Iași, Romania; angela_tecuceanu@yahoo.com (A.T.); dr.anca.sava@gmail.com (A.S.); ruxandravatavu@yahoo.com (R.V.); irina_hm@yahoo.com (I.M.H.J.); andreealudusanu1106@yahoo.com (A.L.); irinabusila@yahoo.com (I.B.); cristi_stan00@yahoo.com (C.I.S.); 2 Department of Plastic Surgery and Burn Unit, “Sf. Spiridon” Emergency Clinical Hospital, 700111 Iaşi, Romania; camelia6@yahoo.com (C.T.); teostamate@gmail.com (T.S.); 3 Department of Pathology, “Prof. Dr. Nicolae Oblu “Emergency Clinical Hospital, 700309 Iaşi, Romania; 4 Department of Plastic Surgery, “Sf. Ioan” Clinical Emergency Hospital for Children, 800487 Galați, Romania; andreeam_c@yahoo.com; 5 Department of Plastic Surgery, “Sf. Ap. Andrei” Emergency Hospital, 800487 Galați, Romania; iulia_dabija@yahoo.com; 6 Department of General Surgery, “Sf. Spiridon” Emergency Clinical Hospital, 700111 Iaşi, Romania; 7 Department of Medical Sciences I, “Grigore T. Popa” University of Medicine and Pharmacy, 700115 Iași, Romania; 8 Department of Ophthalmology, “Grigore T. Popa” University of Medicine and Pharmacy, 700115 Iași, Romania; roxana-elena.ciuntu@umfiasi.ro; 9 Department of Emergency Medicine, “Grigore T. Popa” University of Medicine and Pharmacy, 700115 Iași, Romania; mihaela.corlade2@umfiasi.to

**Keywords:** enzymatic debridement, negative wound pressure therapy, burns

## Abstract

Background: In thermal injuries, enzymatic debridement is a viable option for treating partial- and full-thickness burns, allowing for rapid removal of damaged tissue with minimal bleeding and without sacrificing healthy dermis. Enzymatic debridement using Nexobrid^®^ combined with negative wound pressure therapy (NWPT) appears to promote healing, as enzymatic debridement helps preserve healthy tissue integrity and epithelial reserves. We explored therapeutic alternatives following enzymatic debridement to assess healing outcomes and reduce reliance on skin grafts. Methods: 24 patients with deep or partially deep thermal burns on 5–40% of total body surface area (TBSA) underwent enzymatic debridement; then, half received NWPT and the other half were treated with topicals. Results: Enzymatic debridement effectively removed necrotic tissue and facilitated healing. Only three patients required skin grafts (<10% TBSA). Enzymatic debridement combined with NWPT expedited daily healing, reduced hospitalization days, and eliminated wound secretion, as confirmed by bacteriological examination. This approach was more effective compared to enzymatic debridement followed by topical treatments. Conclusions: Nexobrid^®^ in combination with NWPT is a promising alternative to surgical treatment, improving healing, reducing the need for skin grafts, and alleviating pain associated with dressing changes. It may be particularly useful in extensive burns, where epithelial reserves are limited.

## 1. Introduction

Plastic surgeons prioritize treating burn lesions, recognizing them as some of the most challenging surgical wounds. These injuries are considered a severe form of physical and psychological trauma for any person who sustains them [1].

Burns are classified as either superficial or deep, with direct implications for spontaneous epithelialization. Superficial burns and superficial partial-thickness burns are both considered superficial. These types are characterized by the destruction of the epidermal layers without the basement membrane being affected. By contrast, in deep burns, including in deep partial-thickness burns, the basal membrane is damaged, resulting in a diminished capacity for spontaneous epithelialization, depending also on the surface area of the burn. Third-degree burns are defined by the complete destruction of both the epidermis and dermis, along with varying damage to underlying structures [2,3].

Superficial burns are known to typically heal within 14–21 days with the help of local dressings and topicals, leaving no scars. On the other hand, deep partial- and full-thickness burns often require early surgical excision and grafting as the standard of care (SOC) [2,4,5].

Early debridement and the preservation of viable tissue are generally considered essential for achieving positive functional and esthetic outcomes, but this is challenging in practice. Surgical debridement involves removing both necrotic and viable tissue that is particularly disadvantageous to functional areas, especially in the extremities. A high risk of bleeding and the susceptibility to infections are two immediate concerns following surgical debridement [3]. A single type of dressing that works effectively in every burn situation is not available [6]. Rather, negative wound pressure therapy (NWPT) may provide a more viable option for addressing the specific needs of these wounds [7,8].

Since the Second World War, there have been multiple attempts to develop an enzymatic debridement agent as an alternative to surgical debridement. Studies on bromelain began much earlier, in 1891, when the Venezuelan chemist Vicente Marcano successfully isolated bromelain for the first time by fermenting pineapple fruits. In 1892, Russell Henry Chittenden, assisted by Elliott P. Joslin and Frank Sherman Meara, studied the subject in more detail and named the substance ‘bromelin’. Later, the term ‘bromelain’ was used to denote any protease extracted from the plant family *Bromeliaceae* [9].

The introduction of Nexobrid^®^, in 2013, marked a significant advancement in treating partial to deep burn injuries. Research confirmed the efficacy and specificity of this product containing a concentrated solution of proteolytic enzymes enriched with bromelain from pineapple stems, prompting pilot centers to start using it on sensitive areas such as the face, genitals, and limbs. This was subsequently included as a recommendation in the European Consensus guidelines updated in 2020 [4,5].

Deep burns caused by thermal injuries are particularly complex, featuring mosaic patterns of deep partial burn lesions overlapping full-thickness burns. Previously, these would have been treated with surgical debridement followed by split-thickness skin grafting. The aim of surgical debridement is to remove necrotic tissue until small hemorrhagic areas specific to healthy tissue are obtained. It is performed tangentially, suprafascially or fascially, assessing the vascularization of the underlying healthy tissue to establish optimal excision depth. Depending on the depth of the burn, structures under the dermis may be exposed [3,10,11].

By contrast, enzymatic debridement with Nexobrid^®^ helps preserve the reticular dermis integrity of the skin., which can serve as the foundation for healing through the gradual recolonization of epithelial and endothelial elements, mimicking the role of synthetic substitutes [1,11]. Following enzymatic debridement, using negative pressure therapy can seal the exposed healthy tissue and stimulate the angiogenesis phenomenon through the subatmospheric effect created. This favors spontaneous epithelialization as a result of preserving the cutaneous reserves offered by the enzymatic technique, and it also avoids the need to sacrifice other donor skin areas.

We conducted a prospective clinical study on patients diagnosed with acute thermal burn lesions to explore specific surgical strategies that may improve patient care and outcomes. Our positive experience with negative wound pressure therapy (NWPT) in treating post-traumatic soft tissue defects, in light of existing studies, prompted us to explore this treatment option for burn patients who undergo enzymatic debridement and may not need skin grafts [12].

## 2. Materials and Methods

### 2.1. Study Design and Setting

This prospective clinical study was conducted on 24 patients treated in the Plastic Surgery and Burns Units of “Sf. Spiridon” Emergency Hospital Iași, Romania, from December 2021 to December 2022.

### 2.2. Study Participants

All the patients included in the study were diagnosed with thermal burns caused by flame, hot liquid, or contact, manifesting as partial-thickness, deep partial-thickness, and full-thickness lesions covering 5% to 40% total body surface area (TBSA). Each patient provided written informed consent and agreed to attend regular follow-up appointments. The exclusion criteria were as follows: lack of consent; pregnancy or postpartum period; history of allergies to compounds based on pineapple, papaya, bromelain, or papaverine; corticosteroid therapy; previous use of topical medications containing silver ions in their composition; penetrating burn wounds; cases where foreign materials like implants or pacemakers were or could be exposed during debridement; chemical burns; and wounds contaminated with radioactive and other hazardous substances, to avoid unforeseen reactions with the product.

A total of 24 patients with similar lesions regarding location and TBSA were divided into two groups. Group A consisted of 12 individuals with thermal burn injuries, with a TBSA ranging from 5 to 40%, localized on the front and back of the torso, as well as on the upper and lower limbs. The clinical diagnosis was established based on the “rule of nine”, which considered the surface area of a patient’s palm as 1% of TBSA. For group A, after enzymatic debridement, NWPT was employed to facilitate the healing process. Wounds were dressed using sterile foam secured by staples, and a sterile occlusive transparent film was applied over these areas. A connector was positioned centrally and connected to the NWPT device, setting the pressure to -125 mmHg. The wound was assessed every five days, with repeated application of adjusted NWPT.

In group B, we enrolled 12 patients with similar characteristics to those in group A in terms of affected areas and TBSA, and the same enzymatic debridement protocol was followed. Subsequently, we proceeded with wound treatment using topical ointments, specifically low-molecular-weight hyaluronic acid (LMW-HA) and hyaluronic acid silver powder spray.

### 2.3. Surgical Procedure

While existing studies highlight the advantages of administering enzymatic debridement at the patient’s bedside, our approach entails performing enzymatic debridement in the operating room to minimize the risk of contamination [13].

A note should be made that, prior to enzymatic debridement, it is important to prepare the wound, which we did with a sterile isotonic solution of NaCl 9 mg/mL used for 2 h to keep the lesion moist. Additionally, pain management was initiated at least 15 min before applying the enzymatic agent, and protection of the surrounding tissue was ensured with a sterile gauze barrier impregnated with Vaseline and secured with sutures to prevent the product from slipping.

The first stage of the surgical procedure included clearing away debris and performing local debridement of the soft tissue. Enzymatic debridement came second and consisted in the following steps: (1) shake vial to decompact the sterile powder of proteolytic enzymes enriched with bromelain, (2) unscrew cap of sterile gel, (3) carefully open vial, additionally loosening the powder if necessary using a sterile spatula, (4) add powder to gel composition, (5) mix for one or two minutes until homogeneous, pale yellow to light brown in color, (6) apply within 15 min of its preparation.

We used vials of 5 g powder and 50 g gel which, when mixed, form a homogeneous gel sufficient for covering 250 cm^2^ of burn area. We used it to cover up to 15% TBSA in a layer 1.5–3 mm thick. Then, the wound was covered with a sterile occlusive layer, and a sterile dressing was placed on top. The dressing was kept in place for 4 h and then removed under aseptic conditions. At this point, any dissolved eschar was removed with a sterile spatula until a pink surface with punctiform bleeding or whitish tissue was revealed. A dressing impregnated with an antibacterial solution based on chlorhexidine was then applied for 2 h, and, subsequently, the wound was evaluated after removing Nexobrid^®^. We did not reapply to the same area, regardless of the amount of eschar surface removed.

### 2.4. Assessment of Wound Healing and Pain

To monitor the progression of healing with the two methods (enzymatic debridement and NWPT versus enzymatic debridement and LMW-HA topicals), we measured the wound areas relative to the total skin surface for each patient (calculated based on weight in kg and height in cm), comparing the results of relatively similar burn wounds in terms of size and severity. Due to the variability in the burn surface areas (between 5% and 40%), we tracked the healing progression on burn areas of up to 15% for similar degrees and locations, considering that we applied enzymatic debridement as well as negative pressure therapy on a maximum surface of 15% TBSA per stage. We also sought to compare similar areas because the daily healing rate is normally higher in a burn of 5% than 40% TBSA burn.

The clinical and bacteriological assessment was conducted both on admission and during hospitalization, prompted by ongoing assessment of wound progression, to identify any colonization with Gram-negative or Gram-positive bacteria. During hospitalization, we detected the presence of *Staphylococcus aureus*, *Pseudomonas aeruginosa*, and *Acinetobacter baumannii*.

Additionally, patients completed a series of questionnaires (Visual Analog Scale) to assess the nature and intensity of their pain, where 0 means that the patient does not feel any pain and 10 is classified as unbearable pain.

### 2.5. Statistical Methods

The database was compiled and analyzed in SPSS 20.0 (Statistical Package for the Social Sciences, Chicago, IL, USA). The Shapiro–Wilk test was used to determine the distribution of continuous data, computing mean values ± standard deviation, or frequency percentages for continuous variables with a normal distribution. Independent samples were used to compare continuous variables with the normal distribution using Student’s *t*-test. Results with a *p*-value < 0.05 were deemed statistically significant.

### 2.6. Ethical Approval

The study was conducted in accordance with the Declaration of Helsinki and approved by the Institutional Ethics Committee of “Grigore T. Popa” University of Medicine and Pharmacy, Iași, Romania (131/01.12.2021), and “Sf. Spiridon” Emergency Clinical Hospital, Iaşi, Romania (20/17.03.2021), for studies involving human subjects.

## 3. Results

**Figure 1:**
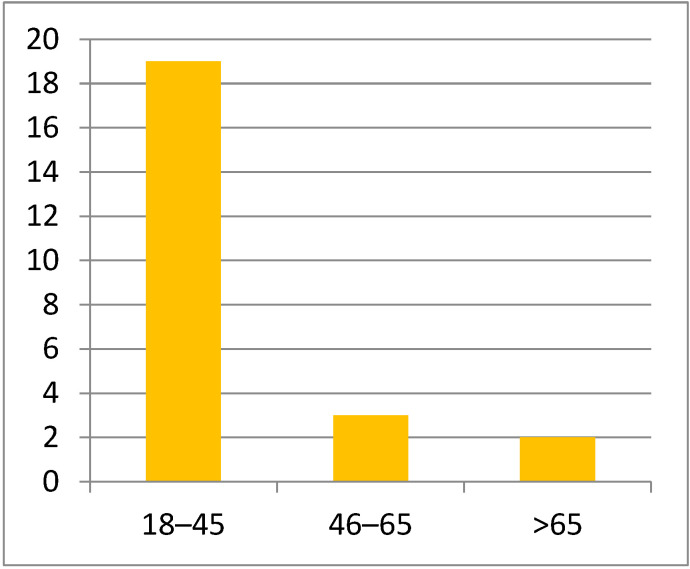
Patient distribution by age.

**Figure 2:**
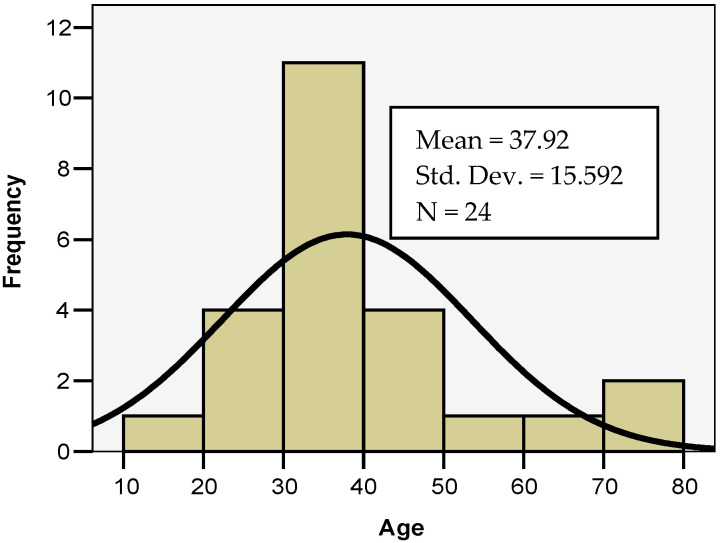
Mean age of study groups.

In both study groups, most patients were men, with a 3:1 male-to-female ratio, probably due to working conditions. Most patients were 18 to 45 years old (19 patients), particularly young adults, emphasizing the significance of the functional and esthetic aspect (Figure 1). The mean age of the study groups was 37.92 ± 15.592 years (Figure 2).

Overall, only five patients were smokers, of whom one reported smoking 25 cigarettes per day. Most patients (17) had at least one associated comorbidity such as diabetes (4), anemia (5), and cardiovascular conditions (8).

Relative to the time of injury, 17 patients (70.83%) sought medical attention within the first 24 h, while 6 patients (25%) did so within 24–72 h. There was only one case of delayed presentation to the hospital (after 72 h).

**Figure 3:**
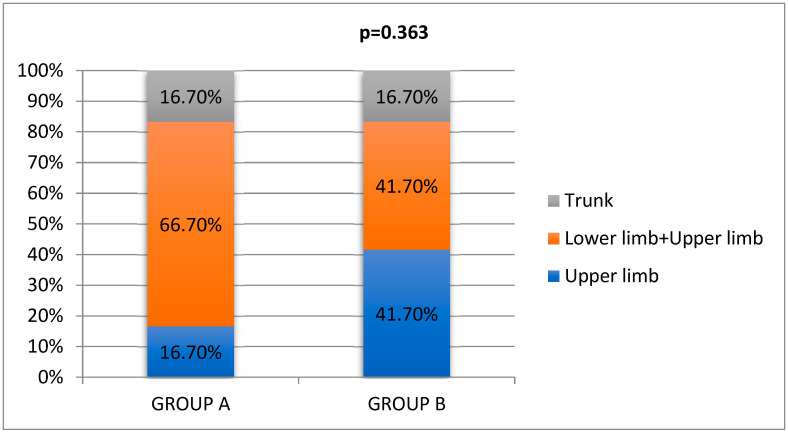
Distribution of burn areas.

In group A, 66.7% of burn injuries were located on both lower and upper limbs (LL + UL), while in group B we identified equal distribution of 41.7% of injuries both on the upper limbs (UL) and on all four limbs (LL + UL) (Figure 3). It is worth noting that all the patients who suffered burns on their lower limbs also had burns on their upper limbs, possibly due to the defense reflex.

**Figure 4:**
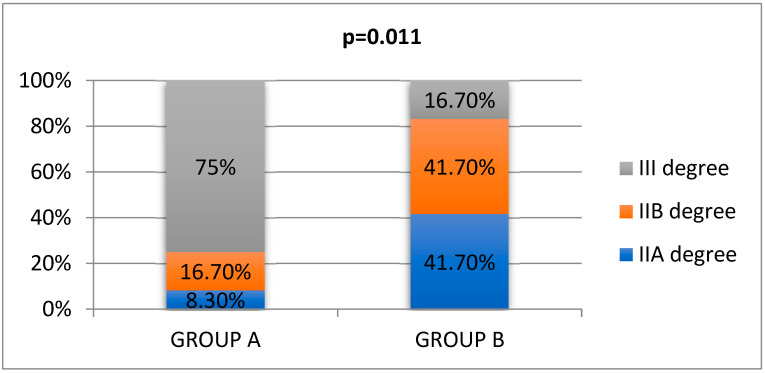
Distribution by burn degrees.

In 54.16% of the cases, patients suffered from second-degree burns, both superficial partial-thickness and deep partial-thickness burns, while 45.83% presented third-degree injuries. Six patients were diagnosed with superficial partial-thickness burns (IIA), seven patients had deep partial-thickness burns (IIB), and eleven patients experienced third-degree burns. The statistical analysis indicated mainly third-degree burns in group A, while in group B the predominant burns were second-degree (Figure 4).

**Figure 5:**
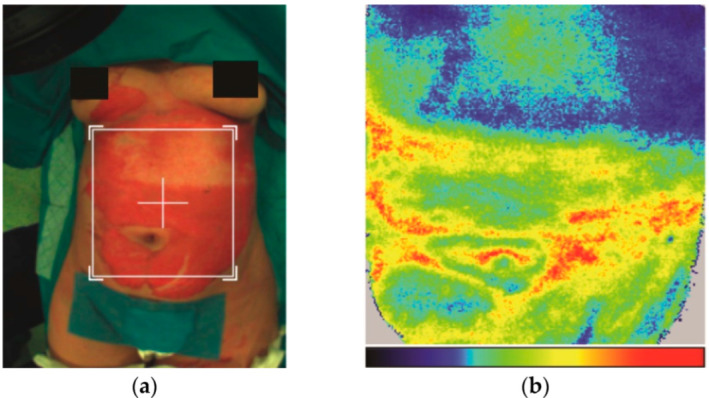
(**a**,**b**) Clinical appearance and laser Doppler imaging of the same area before enzymatic debridement with Nexobrid^®^. Images from the authors’ collection.

The depth of the burn wounds was assessed both before and after enzymatic debridement using a laser Doppler perfusion imaging system to accurately establish the clinical diagnosis as well as to highlight changes in local vascularization after enzymatic debridement. With this method, blue indicates low perfusion of the affected tissue (full thickness), green means low perfusion as well (better than blue), yellow shows moderate perfusion (between superficial partial-thickness and deep partial-thickness burn), and red is indicative of first-degree burns (Figure 5a,b).

**Figure 6:**
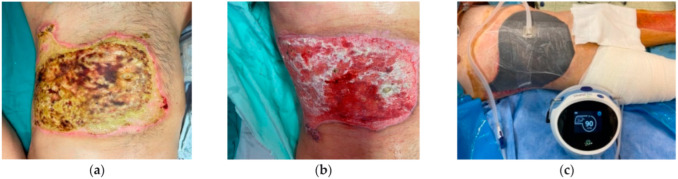
(**a**) Neglected 3rd-degree thermal burn by flame and contact. Day 1 post-admission. (**b**) Enzymatic debridement and two sessions of NWPT. (**c**) Third session of NWPT and mesh graft with 1:6 ratio.

Enzymatic debridement therapy was administered within 1–3 days post-injury for 11 patients, and in one case it was performed 5 days post-trauma. This was the case of the patient presenting after 72 h with third-degree burn injuries on the abdomen and back of the torso (12% TBSA). For this patient, enzymatic debridement was followed by two sessions of vacuum-assisted therapy five days apart, a mesh graft with a 1:6 ratio, and a 3rd NWPT session set to 90 mmHg (Figure 6a–c).

**Figure 7:**
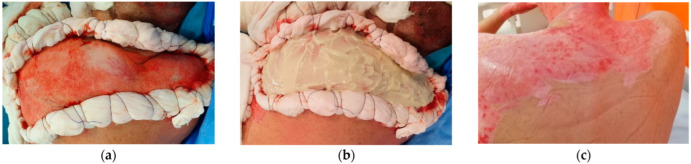
(**a**) IIB-III degree thermal burn by flame. (**b**) Mixture of enzymatic debridement. (**c**) Day 16 after enzymatic debridement and LMW-HA topicals.

**Figure 8:**
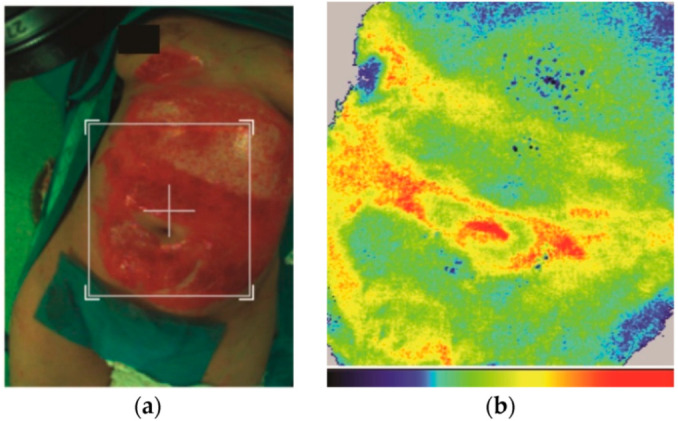
(**a**,**b**) Correlation between clinical appearance and laser Doppler imaging 4 h after applying Nexobrid^®^ Images from authors’ collection.

Enzymatic debridement was performed for all patients, targeting both superficial and deep partial-thickness, as well as full-thickness burn lesions (Figure 7a–c). This approach led to the removal of varying degrees of eschar until the appearance of pinpoint bleeding as an indication of healthy tissue. The efficacy of this treatment was further corroborated by changes in coloration observed during laser Doppler imaging conducted post-debridement. Notably, the deep burn area, initially exhibiting blue coloration, transitioned to green, which signifies improved vascularization compared to the previously blue-colored tissue (Figure 8a,b).

**Figure 9:**
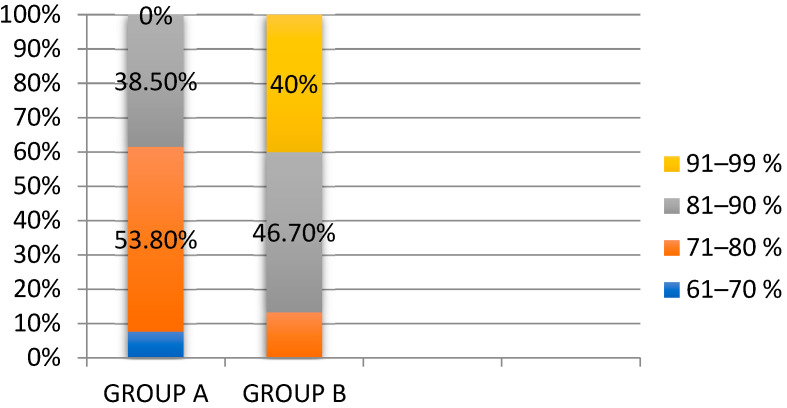
Eschar removal after enzymatic debridement.

In group A, after the application of Nexobrid^®^, 53.8% of patients presented 71–80% eschar removal. Higher rates of 81–90% were achieved in 38.5% of cases, while lower rates of 61–70% were noted in 7.7% of cases (*p* = 0.005). Meanwhile, in group B, 40% of patients achieved 91–99% eschar removal, 46.7% were at 81–90%, and 13.3% presented eschar removal in proportions of 71–80%, as shown in Figure 9.

Based on the amount of eschar removed, the laser Doppler scanning results, the clinical appearance post-debridement, and the burn surface, we decided to use either negative pressure therapy or topical treatment.

**Figure 10:**
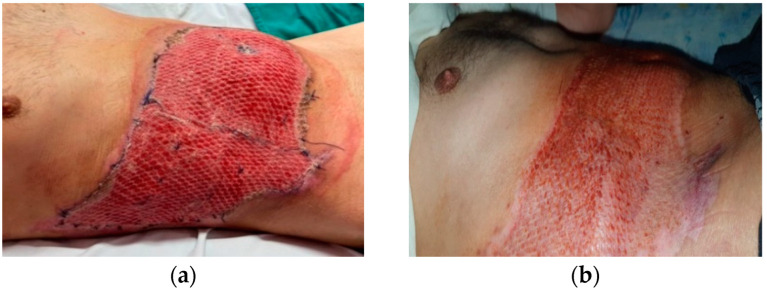
(**a**) 5 days after NWPT and Mesh Graft. (**b**) Follow-up after three months.

Only three patients included in the study, one from group A (Figure 10a,b) and two from group B, required a split-thickness skin graft harvested from the antero-lateral thigh, using electrodermatome with a thickness of 0.2–0.3 mm. These patients achieved complete graft uptake on an area of 8%, 9%, and 10% respectively.

The application and removal of the enzymatic debridement agent was carried out under careful monitoring of vital parameters by the anesthesiologist. The enzymatic debridement agent was used on patients with upper-limb burns under locoregional anesthesia (axillary plexus). For burns located on the lower limb, spinal anesthesia and locoregional anesthesia (sciatic plexus) were preferred, allowing for removal without the need for additional intervention by the anesthesiologist and without any side effects (seven cases).

**Figure 11:**
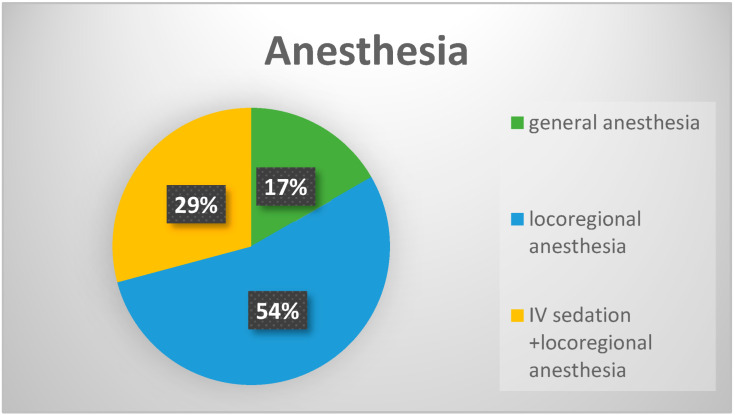
Types of anesthesia used for burns on the trunk, upper, and lower limbs.

For patients with burn injuries on both the upper and lower limbs, we adopted a combined approach using loco-regional anesthesia and intravenous sedation (13 cases). The choice of loco-regional anesthesia was based on the extent and severity of the burns, as indicated in Figure 11. For the lesions situated on the trunk, we decided to perform general anesthesia (four cases). This approach helped us prevent and avoid known complications of general anesthesia.

**Figure 12:**
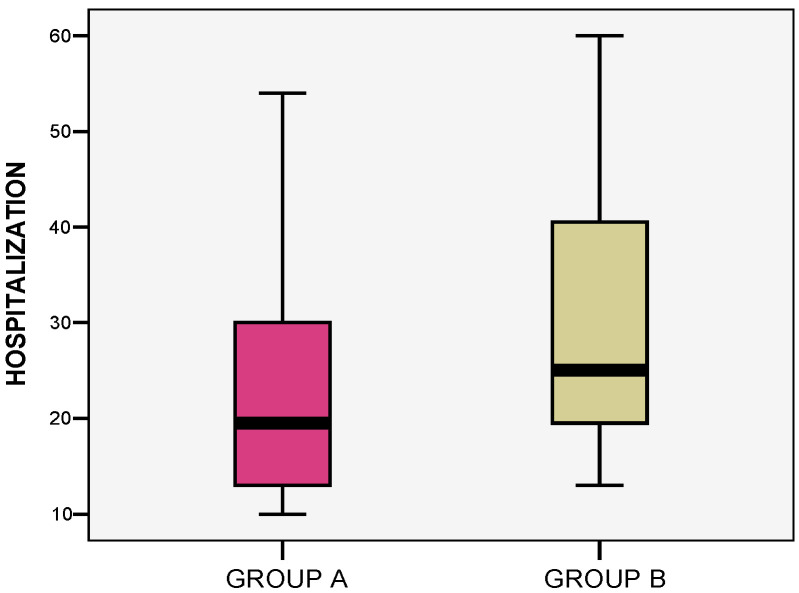
Associations between hospitalization days required for healing for each group.

**Figure 13:**
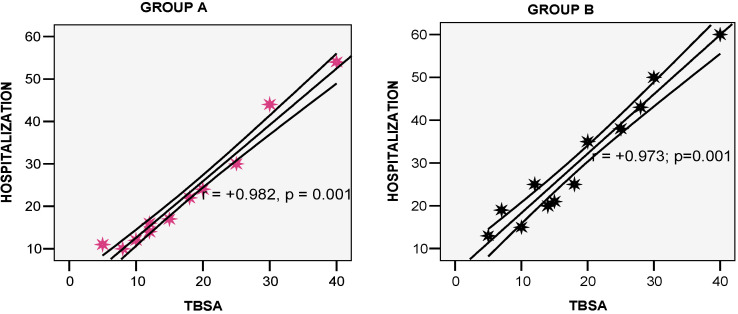
Associations between TBSA and hospitalization in study groups.

Hospitalization duration was analyzed to assess speed of healing. Patients in group A required between 10 and 54 days (mean 23.67 ± 13.81), while the patients in group B spent between 13 and 60 days in hospital (mean 30.33 ± 14.84), as shown in Figure 12. The Student t-test result was non-significant (*p* = 0.267). Strong positive associations were noted between TBSA and the number of hospitalization days in both groups (r = +0.982 at *p* = 0.001, and r = +0.973 at *p* = 0.001, respectively) (Figure 13). Patients with contaminated wounds needed longer healing periods compared to non-contaminated burns, with an average healing time of 36.4 days for group A, and 41.6 days for group B.

The daily rate of healing progression for non-contaminated wounds in group A varied between 2.5% from the initial burn surface area (for a patient with burn surface area of 5820 cm^2^) and a maximum of 10% (in the cases of two patients with similar burn surface areas: 1432 cm^2^, amounting to 8% TBSA, and 2050 cm^2^, equivalent to 10% TBSA, respectively) with an average of 5.67%. For group B, we recorded a minimum of 2% daily healing progress in a patient with a burn surface area of 4950 cm^2^ and a maximum of 7.69% in a patient with a burn surface area of 1045 cm^2^, the group average being 4.06% per day.

**Figure 14:**
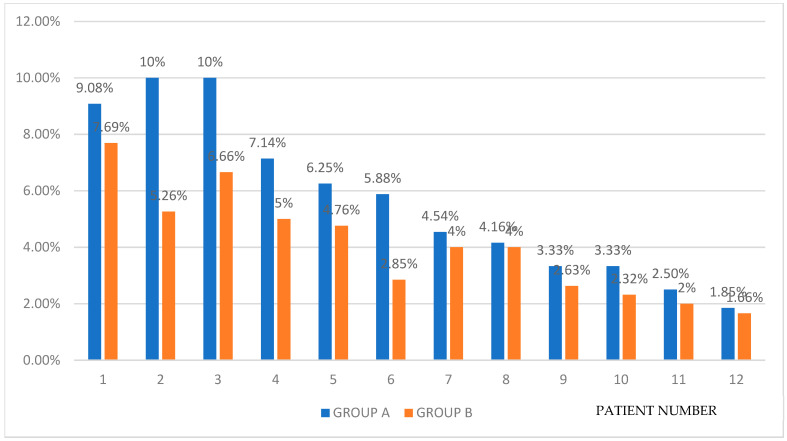
The daily healing progression rate from the initial burn injury.

Relative to the smallest initial burn surface area included in the study (5%), we found a daily burn healing rate in group A of 9.08% and of 7.69% in group B. At the other end of the spectrum, relative to the largest initial surface area (40%), we calculated a daily healing rate of 4.54% in group A and 3.33% in group B; the reference used for calculating healing rates was 15% TBSA (Figure 14).

On admission, *Pseudomonas aeruginosa* was found in two patients with burns over 10% who presented 72 h post-trauma, and *Staphylococcus aureus* in another two patients. There were five contaminated wounds in each study group (five with *Staphylococcus aureus*, three with *Pseudomonas aeruginosa*, and two with *Acinetobacter baumannii*). We administered antibiotic treatment according to the antibiogram. Locally, in group A, we achieved negative bacteriological secretion after removing the second NWPT session and complete debridement of the eschar. In group B, in addition to general treatment, we performed daily local wound care using chlorhexidine-based antiseptics and LMHA-topical with silver powder spray until the results were negative. For group A, we achieved wound negativity within 10 days on the surfaces where we used enzymatic debridement followed by NWPT, while in group B, positive values persisted for up to 25 days.

Physiotherapy was performed for each affected area, starting on the first day after treatment. We monitored patient progress daily in group B. In these cases, we used the “wet-to-dry” dressing method to address desiccation, along with LMW-HA topicals and hyaluronic acid silver powder spray (for contaminated wounds) and documented the cases by taking digital photographs. For patients included in group A, the clinical evaluation of the wound was performed every 5 days, with the negative pressure therapy being reapplied in all 12 cases.

Each patient underwent comprehensive hydroelectrolytic rebalancing treatment, pain management, antithrombotic prevention, and physical therapy. Before discharging each patient, we collected bacteriological samples to confirm the negative result.

Regarding the patients’ assessment of their pain with the Visual Analog Scale (VAS), patients selected a picture that best represented their level of pain, and that was translated into scores. Overall, the average pain intensity score was 4.54. Patients in group A reported less pain compared to those in group B. All responses in group B to the Visual Analog Scale (VAS) were over 5.

Only one patient who received enzymatic debridement treatment simultaneously for upper and lower limbs in the same session reported burning sensations immediately after applying the agent, and a higher dose of opioids was administered to alleviate his pain.

The pain management protocol employed includes the systematic use of opioids, along with analgesia, nonsteroidal anti-inflammatory drugs, and anxiolytics as adjuvants (Table 1). Due to the variability in patient responses, predicting the exact dosage required to achieve a certain level of sedation was challenging.

In our study, 54% of the patients received intravenous sedation that was performed with opioids combined with analgesics and nonsteroidal anti-inflammatory drugs during enzymatic debridement and post-procedurally.

After discharge, not all patients received compression garments or physiotherapy due to cost concerns, which could impact long-term functional and esthetic outcomes.

## 4. Discussion

The early removal of eschar following a deep burn injury is regarded as the most effective approach, as it offers significant advantages for the healing process and aids in the prevention of complications associated with scarring [14]. While surgical methods of early excision and grafting represent the standard of care for deep burns, they are frequently accompanied by various complications, a high degree of invasiveness, and the potential for substantial bleeding [12,15]. Achauer and Sood [14] emphasized the importance of early treatment due to the significant blood loss during surgical excision of the burned area, as indicated in Table 2.

Nexobrid^®^ is the only enzymatic agent that has received FDA approval for treating severe thermal burns, first for adults, in 2022, and later for pediatric patients, in 2024. In recent years, there has been a significant increase in the use of enzymatic debridement as an alternative to surgical treatment for deep burns [6,7,16,17]. The primary goal of enzymatic debridement is to achieve selective removal of damaged tissue while preserving healthy tissue, particularly the cutaneous epithelial reserves that are essential for promoting healing, and our findings support that.

Moreover, negative wound pressure therapy (NWPT), also known as vacuum-assisted wound closure (VAC), has become a valuable adjunct to enzymatic debridement in managing deep burns. It has played a critical role in the reduction in bacterial load in burn wounds [6]. This type of outcome was confirmed through our bacteriological examinations conducted after the completion of the second session of negative pressure therapy, which lasted 10 days in all 12 cases. By contrast, patients in group B achieved a negative bacteriological result, with an average of 25 days.

Although we introduced this technology in our practice with the initial intention to manage non-thermal traumatic wounds and pressure ulcers, NPWT proves to be an invaluable complement to enzymatic debridement through its multifaceted mechanisms of action. Other evidence shows that NPWT can be combined with a skin graft in burn patients. Negative pressure dressing can improve graft take, speed up the process, and reduce the duration of graft dressings compared to conventional dressing covered with Vaseline gauze alone, as our results also confirm (Figure 7a,b) [8].

We noted that both study groups showed a reduced need for grafting. Only 12.5% of patients required skin grafts, covering less than 10% of the TBSA for each patient, and we observed 100% integration on day 5 by associating the skin graft with NWPT maintained at −90 mmHg (Figure 10a). Comparing burn areas of a similar size between the two groups, the significant prevalence of third-degree burns in group A was evident. This observation further supports the rationale for utilizing negative pressure wound therapy (NWPT) in these cases.

We also recorded that the combination of Nexobrid^®^ with negative pressure wound therapy has proven effective in addressing persistent necrotic tissue, as we did not achieve 100% eschar removal in any patient after enzymatic debridement. Additionally, the choice of ointment base and cover dressing can further facilitate autolytic debridement.

Enzymatic debridement, also known as the minimal-invasive method (MIM), is valued for its ability to preserve the dermis intact, reducing the necessity for surgical debridement and skin grafts [18,19]. While enzymatic debridement is easy to apply, its correct application entails a learning curve to avoid technical errors and prevent unnecessary surgical debridement [20,21,22]. Proper evaluation of the wound before and after application is crucial to accurately determine burn depth and identify the best treatment approach. Skin grafting is necessary when fat exposure is evident, while whitish areas with pinpoint bleeding have a good chance of healing spontaneously, using conservative methods [19,23].

Another notable advantage that we observed is the lower number of hospitalization days compared to the technique associated with enzymatic debridement followed by topicals. In addition, our findings indicate that the combination of Nexobrid^®^ and NWPT results in less pain for the patient than enzymatic debridement followed by topicals, likely due to fewer dressing changes and reduced discomfort during the procedure. The fact that 54% of patients were able to benefit from enzymatic debridement under loco-regional anesthesia, combined with intravenous sedation, is an encouraging development. We observed a considerable reduction in pain, preferring a multimodal strategy, with the aim of keeping the duration and level of sedation as brief and shallow as possible.

In our practice, we introduced laser Doppler imaging to increase the accuracy of diagnosing intermediate burns, which helped us in making effective therapeutic decisions, considered the only FDA-approved technique for evaluating burns. While red areas on the scan indicate superficial burns, blue areas signify deep burns. Yellow areas are still under consideration for conservative treatment, whereas yellow areas combined with green will need a longer healing time. The correlation between the obtained images and the clinician’s experience continues to be the preferred method for approaching burn treatment [24].

The NPWT technique is gaining traction, with recent studies exploring its potential beyond the early trend of using it to treat burns in children. Although the latest Cochrane review from 2019 remains neutral on its effectiveness compared to standard dressings, numerous subsequent studies, including ours, have demonstrated encouraging results [6,25]. In conclusion, the positive outcomes observed in the treatment of deep burn injuries affecting the trunk, upper limbs, and lower limbs, coupled with recent research advancements, encourage us to implement the enzymatic debridement technique in areas with unique anatomical features such as the face, scalp, and genitals.

### Study Limitations

Although the use of negative pressure therapy after enzymatic debridement is a valuable option for burn treatment, the small number of patients included in this study can be considered a limitation, requiring further studies on larger cohorts. Another limitation is the relatively short follow-up period, with most patients being monitored for up to 12 months postoperatively. It would be interesting to observe the functional and esthetic aspects over a 10-year period, as well as the social reintegration of patients with extensive burns.

## 5. Conclusions

Although no ideal treatment exists for burn patients, ongoing efforts aim to develop lifesaving solutions that improve survival chances and achieve functional and esthetic results superior to standard treatment. The efficiency of combined methods surpasses single therapies and can serve as a benchmark in the treatment of this complex pathology.

This study contributes to the ongoing search for optimal therapeutic approaches involving enzymatic debridement, NWPT, and hyaluronic acid topicals to complement the SOC for burn patients, whose injuries inflict substantial physical and psychological suffering. The results of our work offer an optimistic perspective of reduced healing times and avoiding the sacrifice of donor areas, thereby preventing the creation of new wounds that require healing, as well as the formation of additional scars. Avoiding extensive surgical debridement can bring real benefits to the patient.

Cutting-edge technology like laser Doppler imaging is instrumental to more accurate diagnosis of intermediate burn injuries. The preservation of epithelial resources represents an important step in improving outcomes offered by enzymatic debridement, which clinically translates to increased survival chances and enhanced quality of life for these patients. Our study shows that combined treatments yield good results, making it important to adapt modern methods to the needs of each patient.

## Figures and Tables

**Table 1:** Pain management.

	Medication	
Fifteen minutes before applying enzymatic debridement agent	Sedatives	Midazolam iv. Propofol iv.
	Analgezia	Fentanyl iv Morphine iv. Paracetamol iv. Ketoprofen
Upon removal of enzymatic debridement mixture	Sedatives	Midazolam iv. Propofol iv.
	Analgezia	Fentanyl iv Morphine iv. Paracetamol iv. Ketoprofen

**Table 2:** Blood loss during surgical excision relative to time of intervention [14].

Surgical Excision	Blood Loss cc/cm^2^
<24 h	0.45 cc/cm^2^
1–3 days	0.65 cc/cm^2^
2–16 days	0.75 cc/cm^2^
>16 days	0.5–0.75 cc/cm^2^
Infected burns	1–1.25 cc/cm^2^

## Data Availability

The data that support the findings of this study are available from the corresponding author upon reasonable request.
